# Advanced visual network and cerebellar hyperresponsiveness to trigeminal nociception in migraine with aura

**DOI:** 10.1186/s10194-019-1002-3

**Published:** 2019-05-03

**Authors:** Antonio Russo, Alessandro Tessitore, Marcello Silvestro, Federica Di Nardo, Francesca Trojsi, Teresa Del Santo, Rosa De Micco, Fabrizio Esposito, Gioacchino Tedeschi

**Affiliations:** 1 Headache Center, Department of Medical, Surgical, Neurological, Metabolic and Aging Sciences, University of Campania “Luigi Vanvitelli”, Piazza Miraglia 2, I-80138 Naples, Italy; 2 MRI Research Centre SUN-FISM, University of Campania, “Luigi Vanvitelli”, Caserta, Italy; 3 Institute for Diagnosis and Care ‘Hermitage-Capodimonte’, Naples, Italy; 4 Department of Medicine, Surgery and Dentistry, Scuola Medica Salernitana, University of Salerno, Fisciano, Italy

**Keywords:** Migraine, fMRI, Aura, CHEPS, Cerebellum

## Abstract

**Background:**

Despite the growing body of advanced studies investigating the neuronal correlates of pain processing in patients with migraine without aura (MwoA), only few similar studies have been conducted in patients with migraine with aura (MwA). Therefore, we aimed to explore the functional brain response to trigeminal noxious heat stimulation in patients with MwA.

**Methods:**

Seventeen patients with MwA and 15 age- and sex-matched healthy controls (HC) underwent whole-brain blood oxygen level–dependent (BOLD) fMRI during trigeminal noxious heat stimulation. To examine the specificity of any observed differences between patients with MwA and HC, the functional response of neural pathways to trigeminal noxious heat stimulation in patients with MwA was compared with 18 patients with MwoA. Secondary analyses investigated the correlations between BOLD signal changes and clinical parameters of migraine severity.

**Results:**

We observed a robust cortical and subcortical pattern of BOLD response to trigeminal noxious heat stimulation across all participants. Patients with MwA showed a significantly increased activity in higher cortical areas known to be part of a distributed network involved in advanced visual processing, including lingual gyrus, inferior parietal lobule, inferior frontal gyrus and medial frontal gyrus. Moreover, a significantly greater cerebellar activation was observed in patients with MwA when compared with both patients with MwA and HC. Interestingly, no correlations were found between migraine severity parameters and magnitude of BOLD response in patients with MwA.

**Conclusion:**

Our findings, characterized by abnormal visual pathway response to trigeminal noxious heat stimulation, support the role of a functional integration between visual and trigeminal pain networks in the pathophysiological mechanisms underlying migraine with aura. Moreover, they expand the concept of “neurolimbic-pain network” as a model of MwoA including both limbic dysfunction and cortical dys-excitability. Indeed, we suggest a model of “neurolimbic-visual-pain network” in MwA patients, characterized by dysfunctional correlations between pain-modulating circuits not only with the cortical limbic areas but with advanced visual areas as well. Furthermore, the abnormal cerebellar response to trigeminal noxious heat stimulation may suggest a dysfunctional cerebellar inhibitory control on thalamic sensory gating, impinging on the advanced visual processing cortical areas in patients with MwA.

**Electronic supplementary material:**

The online version of this article (10.1186/s10194-019-1002-3) contains supplementary material, which is available to authorized users.

## Introduction

Visual aura is the most common transient neurological symptom experienced by patients during attacks of the so-called “migraine with aura” [1].

In the last decades, the relationship between the aura phenomenon, most likely underlied by cortical spreading depression (CSD), and trigeminal nociceptive pathway activation, subtending headache attacks, has been widely debated [1].

Preclinical evidence strongly supported that CSD-related up-regulation of c-fos expression in trigeminal nucleus caudalis, local release of pro-nociceptive molecules and thalamic activation may modulate the nociceptive inputs to the cortex [2, 3]. However, despite the growing body of functional neuroimaging and electrophysiological studies investigating the neuronal correlates of pain processing in patients with migraine without aura (MwoA) [4], to our knowledge, no similar studies have been conducted in patients with migraine with aura (MwA).

Therefore, in the present study we aimed to explore the functional brain response to trigeminal heat stimulation (THS), using whole-brain blood oxygen level-dependent (BOLD)-fMRI, in patients with MwA compared to healthy controls (HC). To examine the specificity of any observed differences between patients with MwA and HC, we further studied a group of age and sex-matched patients with MwoA.

We hypothesized that cerebral regions involved in visual aura phenomenon would be differentially responsive between patients with MwA and HC. We further hypothesized that functional changes associated with visual aura phenomenon would be present in patients with MwA but not in those with MwoA.

## Materials and methods

### Subject population

According to the criteria of International Classification of Headache Disorders (ICHD-3 beta version) for ‘typical aura with headache’ [5], 20 consecutive MwA patients (experiencing exclusively migraine attacks with visual aura) were prospectively recruited from those referring to the Headache Centre of the University Hospital of Naples. MwA patients with photophobia or visual phenomena during the interictal period were not enrolled. At the same time, 20 consecutive patients with episodic MwoA were recruited. The patients, right-handed and with a normal neurological examination, had never taken migraine preventive drugs in the course of their life. Patients with any other type of headache, somatic or psychiatric conditions (assessed with “Hamilton Depression Rating Scale” and “Hamilton Anxiety Rating Scale”) or intake of daily medication were excluded. Photophobia and visual phenomena has been assessed by an extensive patient interview, specifically oriented to these symptoms.

To avoid any possible aura, migraine, photophobia or pharmacologically related interferences on BOLD signal fluctuation, patients were aura, migraine and photophobia-free and not taking rescue medications for at least 3 days before scanning. In the same way, patients were interviewed 3 days after the MRI scan to exclude patients experiencing a migraine attack in the time frame. Among all patients, three were excluded for a migraine attack during the three-days follow-up period and two patients have been excluded from final statistical analyses due to motion-related signal artefacts. Finally, 17 patients with MwA and 18 patients with MwoA were included in the final analyses. Demographic data, disease duration, migraine attacks (with or without aura) frequency (days/month), and average of pain intensity of migraine (by a visual analogue scale) were obtained from the patients. All patients fill-in the Migraine Disability Assessment Scale (MIDAS) and the Headache Impact Test (HIT-6). Clinical parameters were not different between the MwA and MwoA migraine groups, with the exception of a lower migraine attacks frequency and MIDAS scores in MwA patients, as expected in migraine population. Fifteen age- and sex-matched, right-handed non-migraineurs subjects, without family history of migraine, were recruited as HC (see Table 1 for demographic and clinical features) via advertisements placed in the hospital (e.g. posters and flyers), via word-of-mouth referrals, and from a database of research volunteers maintained by the MRI Research Centre of the University Hospital of Naples. All subjects underwent a preliminary MRI examination, to exclude any relevant structural abnormality.

**Table 1:** Clinical characteristics of patients with MwoA, patients with MwA and HC

Parameter	Group	Mean ± SE	*p* value
Gender	MwoA	8 M; 10F	0.85^a^
	MwA	7 M; 10F	0.66^b^
	HC	5 M; 10F	0.53^c^
Age (years)	MwoA	30.05 ± 1.32	0.30^a^
	MwA	32.47 ± 2.01	0.06^b^
	HC	27.40 ± 1.53	0.18^c^
Disease duration (years)	MwoA	8.38 ± 1.66	0.45
	MwA	10.41 ± 2.24	
Frequency (days/month)	MwoA	5.61 ± 0.80	< 0.001
	MwA	1.00 ± 0.25	
MIDAS	MwoA	19.61 ± 2.61	0.002
	MwA	7.53 ± 1.50	
HIT-6	MwoA	62.11 ± 1.52	0.06
	MwA	57.82 ± 1.71	
HAM-D	MwoA	5,11 ± 0,82	0.18
	MwA	4,79 ± 0,76	
HAM-A	MwoA	5,71 ± 0,96	0.28
	MwA	5,34 ± 0,89	
VAS of attack intensity	MwoA	7.79 ± 0.25	0.50
	MwA	7.56 ± 1.49	
Side of THS	MwoA	9 R; 9 L	0.85^a^
	MwA	9 R; 8 L	0.73^b^
	HC	7 R; 8 L	0.85^c^
NRS during THS at 51 °C	MwoA	4.5 ± 0.60	0.61^a^
	MwA	4.94 ± 0.65	0.89 ^b^
	HC	5.01 ± 0.61	0.50 ^c^

*MwoA* patients with migraine without aura, *MwA* patients with migraine with aura, *HC* healthy controls, *M* male, *F* female, *MIDAS* migraine disability assessment scale, *HIT-6* headache impact test, *HAM-D* Hamilton depression rating scale, *HAM-A* Hamilton anxiety rating scale, *VAS* visual analogic scale, *THS* trigeminal heat stimulation, *R* right side, *L* left side, *NRS* numerical rating scale

^a^MwoA vs MwA. ^b^MwA vs HC. ^c^MwoA vs HC

### Standard protocol approvals, registrations, and patient consents

The experiments conformed to the principles of the Declaration of Helsinki and were approved by the ethics committee of the University of Campania “Luigi Vanvitelli”. All participants provided informed written consent after the experimental procedure had been explained.

#### Trigeminal heat stimulation

THS were conducted by the contact heat evoked potential stimulator (CHEPS) (Medoc Ltd., Ramat Yishai, Israel), a MRI-compatible thermode, ending with a surface of a heating thermo-foil of 572.5 mm^2^ (Minco Products, Inc., Minneapolis, MN) covered with a 25 μm layer of thermos-conductive plastic (Kapton®, thermal conductivity at 23 °C of 0.1–0.35 W/m/K). By means of CHEPS very high temperatures can be rapidly reached (up to 70 °C/s).

THS are applied on the cheek corresponding to the side of the head more frequently involved by headache attacks in migraine patients whereas the side of the face in HC were stimulated with regard of the side of the face tested in migraine patients, to match right and left sides of the faces in to the three experimental groups.

We administered mildly painful stimuli at 41 °C and moderately painful stimuli at 51 °C [6] with a random modality, in order to overcome both habituation and expectation phenomena. The personnel who performed the scans and the THS was blinded to subject status.

#### Functional magnetic resonance imaging parameters and pre-processing

The fMRI imaging and pre-processing parameters employed in the present experiment has been extensively delineated in our previous works [6, 7].

MRI was performed on a 3-T scanner (Signa HDxt, GE Healthcare, USA) with an 8-channel parallel head coil. Each fMRI scan consisted of 300 volumes of a repeated gradient-echo echo planar imaging sequence. Three-dimensional T_1_-weighted images (FSPGR BRAVO sequence) and T_2_-fluid-attenuated inversion recovery (T_2_-FLAIR) sequence was also performed.

Functional image time-courses processing was conducted employing the software BrainVoyager QX (Brain Innovation, The Netherlands). All the scans were re-aligned to the first included volume scan using a Levenberg–Marquardt algorithm to perform a correction of movement artefacts. After that, the motion parameters were accurately investigated to check the presence of compromising residual motion (> 1 functional voxel).

Based on the images registered using three-dimensional anatomical scans, the functional image-time series were warped into Talairach space and resampled into 3-mm isotropic voxel time series. After all, the resampled volume time series were spatially smoothed with a 6-mm full-width-at-half-maximum Gaussian kernel size to conduct a group-level analysis.

### fMRI

#### Experimental protocol

Each fMRI session was constituted by consecutive sub-sessions in which migraine patients and HC underwent two different THS stimuli (41° and 51 °C) according to a previously reported event-related experimental design [6, 7]. During each fMRI sub-session, jitters of 41° or 51 °C THS (each lasting 600 ms) was applied on the maxillary area, in a random modality, with a jittered inter-stimulus interval of 14 ± 1 s (*total session duration 5 min 17 s*).

Before the fMRI recordings, out of the scan, the characteristics of the applied thermal heat stimuli were widely elucidated to migraine patients and HC. During each fMRI scan, immediately after each jitter of 41° and 51 °C stimulations, subjects verbally rated the intensity of perceived pain intensity on a numerical rating scale (NRS) ranging from 0 (“no pain”) to 10 (“worst pain imaginable”). During fMRI sessions, patients were monitored for physiological parameters and absolute movement inside the scanner (see Additional file 1).

#### Statistical analysis

The description of statistical analysis used in the present study were carefully described in our previous works [6, 7]. The variance of all image time series was estimated voxel-wise by a random-effects convolution-based general linear model analysis [8]. For each subject, the two fMRI time series corresponding to the two different sub-sessions were temporally normalized to z scores and concatenated before entering the General-linear-model (GLM) fitting (see below). Two “event-type” GLM predictors of interest encoding the responses to the two THS (41° and 51 °C) were defined using the double-gamma function as hemodynamic input function for the linear convolution. For each subject and voxel included in the slab of imaging, the “beta” weights of all repressors were predicted by a GLM fit-refit approach.

Single group contrasts were used to map, for each subjects group, the whole brain distribution of the areas involved in activation following the external stimulus (*p* = 0.001 cluster-level corrected) during THS at 41 °C and 51 °C.

To extract population-level inferences from statistical maps, the two beta estimates for the predictors of interest at each voxel entered a second-level analysis of variance considering the *aura phenomenon* as random observations (random effects analysis of variance ANOVA). A two-way ANOVA table was calculated, with one within-subject factor correlated to the “temperature” effect and one between-subjects factor correlated to the *migraine* effect. From the computed ANOVA tables, contrast t-maps for the main effects of THS as well as for the two-group and one-group (MwA > MwoA) differential effects were calculated and loaded in the Talairach-normalized high-resolution “Colin-27” template.

To identify the brain regions with statistically significant effects, a threshold was considered to the t-maps, to preserve against false-positive voxels at 5% (multiple comparisons correction).

In order to correct functional clusters for multiple comparisons, a cluster-level threshold was applied to the maps that protected against false-positive clusters at 5% (cluster-level corrected for multiple comparisons). More specifically, from an (uncorrected) voxel-level threshold of *p* = 0.001, a whole-brain correction approach based on Monte Carlo simulations was applied to identify the minimum cluster size. In order to exclude a confounding effect on results, analysis of covariance (ANCOVA) has been conducted, including age and gender as nuisance variables. In all patients (as a group) and in the MwA and MwoA patients (considered as two subgroups), the correlations between regional BOLD responses to THS and the clinical features (i.e. disease duration, migraine frequency, average of pain intensity of migraine attacks, scores of MIDAS, HIT-6 scores and perceived pain intensity of experimental stimuli) were calculated using Pearson correlation coefficients. Then the Bonferroni correction for multiple comparison was applied.

#### Data availability

Clinical, neuroimaging and statistical data will be available upon request from any qualified investigator.

## Results

### Behavioural data (pain ratings)

Pain intensity ratings (by NRS) were not significantly different between patients with MwA, HC and patients with MwoA at any level of experimental stimuli. (NRS ± SE at 41° MwA: 2.17 ± 0.46, HC: 3.67 ± 0.52, MwoA: 2.94 ± 0.70; MwA vs HC *p*:0.071, MwA vs MwoA *p*:0.27, MwoA vs HC *p*:0.39) (NRS ± SE at 51° MwA: 4.94 ± 0.65, HC: 5.01 ± 0.61, MwoA: 4.5 ± 0.60; MwA vs HC *p*:0.89, MwA vs MwoA *p*:0.61, MwoA vs HC p:0.50).

### Imaging fMRI data

During THS at two different intensities, functional changes were identified in the three groups in several regions known to be involved in pain processing (*p* < 0.001 cluster level corrected) [9]. During the mildly painful THS (41 °C), no differences in BOLD-response were observed between patients with MwA, HC and patients with MwoA.

**Fig. 1:**
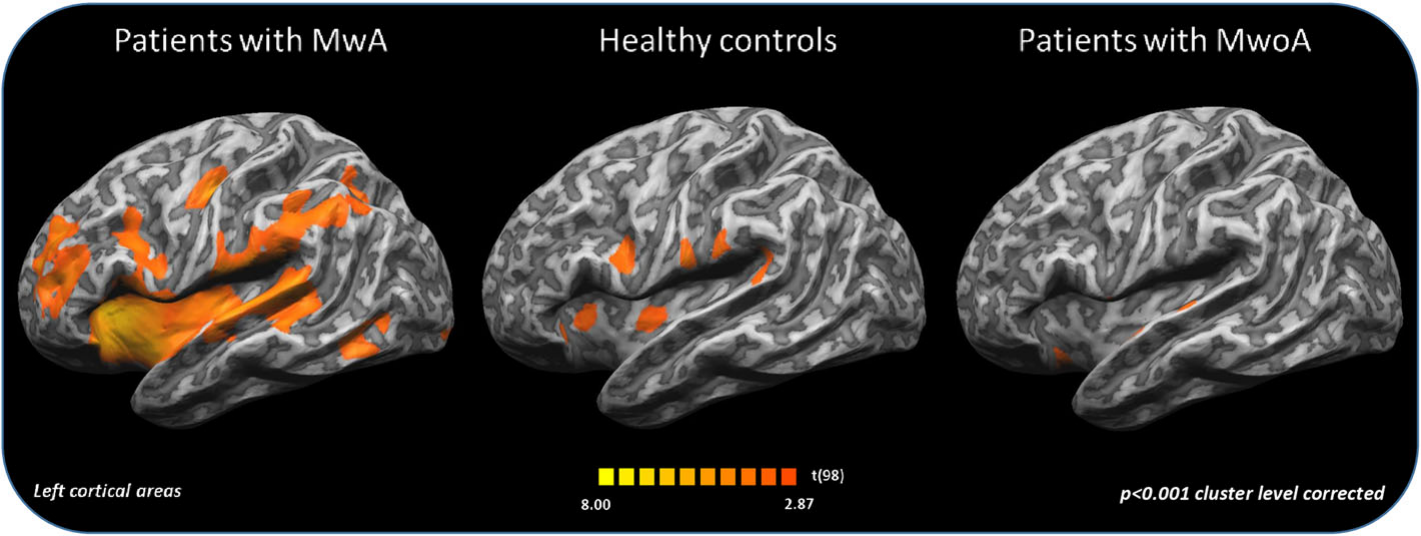
Group-level (main effects) BOLD-response of advanced visual network (encompassing lingual gyrus, inferior parietal lobule, inferior frontal gyrus and medial frontal gyrus) in patients with MwA, MwoA and HC during THS at 51 °C. Statistical maps were obtained overlaying an inflated 3D brain surface from the ‘Colin 27’ atlas (*p* < 0.001 cluster-level corrected). MwA = migraine with aura; MwoA = migraine without aura; HC = healthy control)

**Fig. 2:**
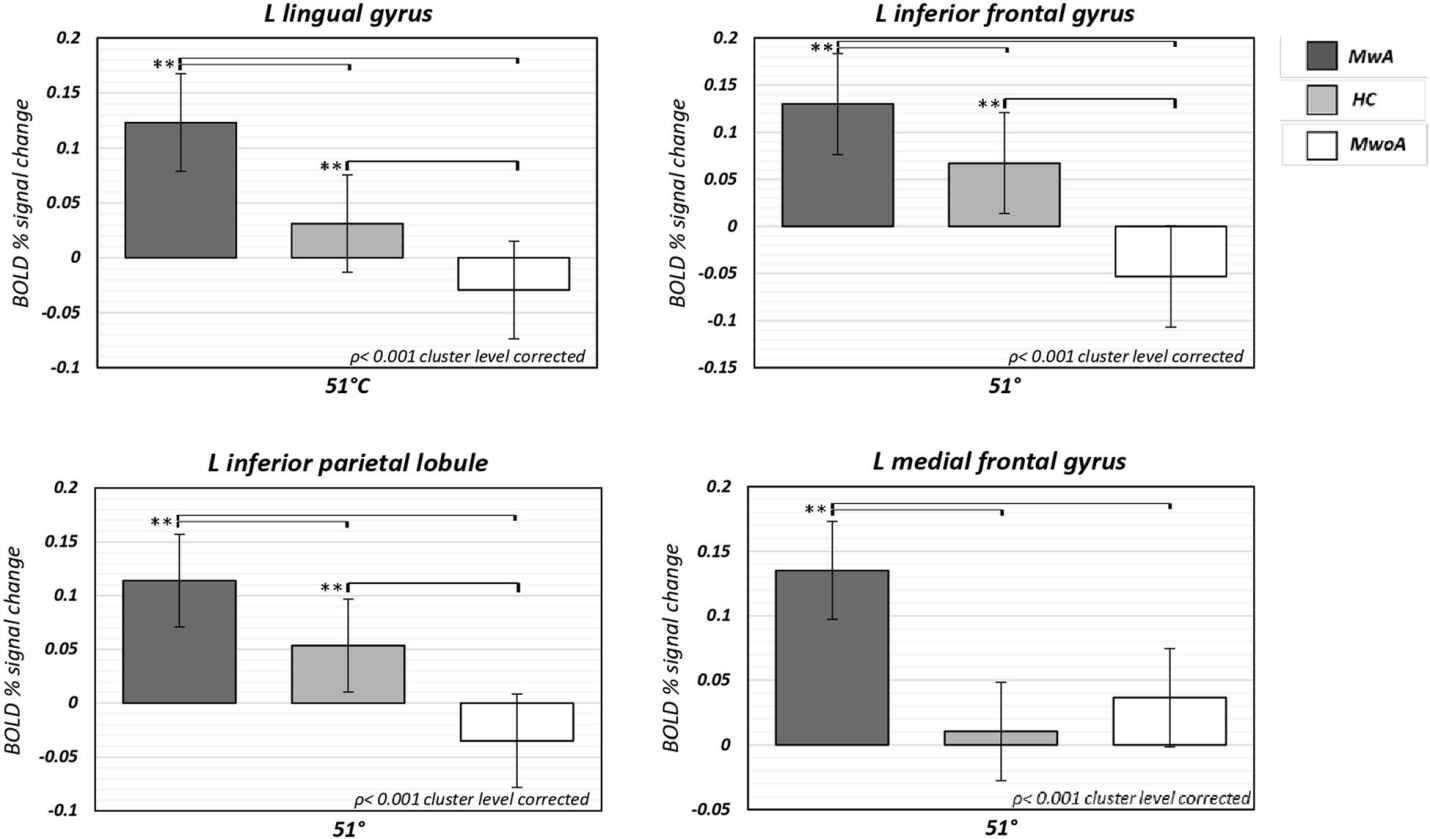
Percent of BOLD signal change (mean ± SE) at 51 °C from left lingual gyrus, left inferior parietal lobule, left inferior frontal gyrus and left medial frontal gyrus clusters in patients with MwA compared to HC and patients with MwoA. SE = standard error; MwA = migraine with aura; HC = healthy controls; MwoA = migraine without aura.

**Fig. 3:**
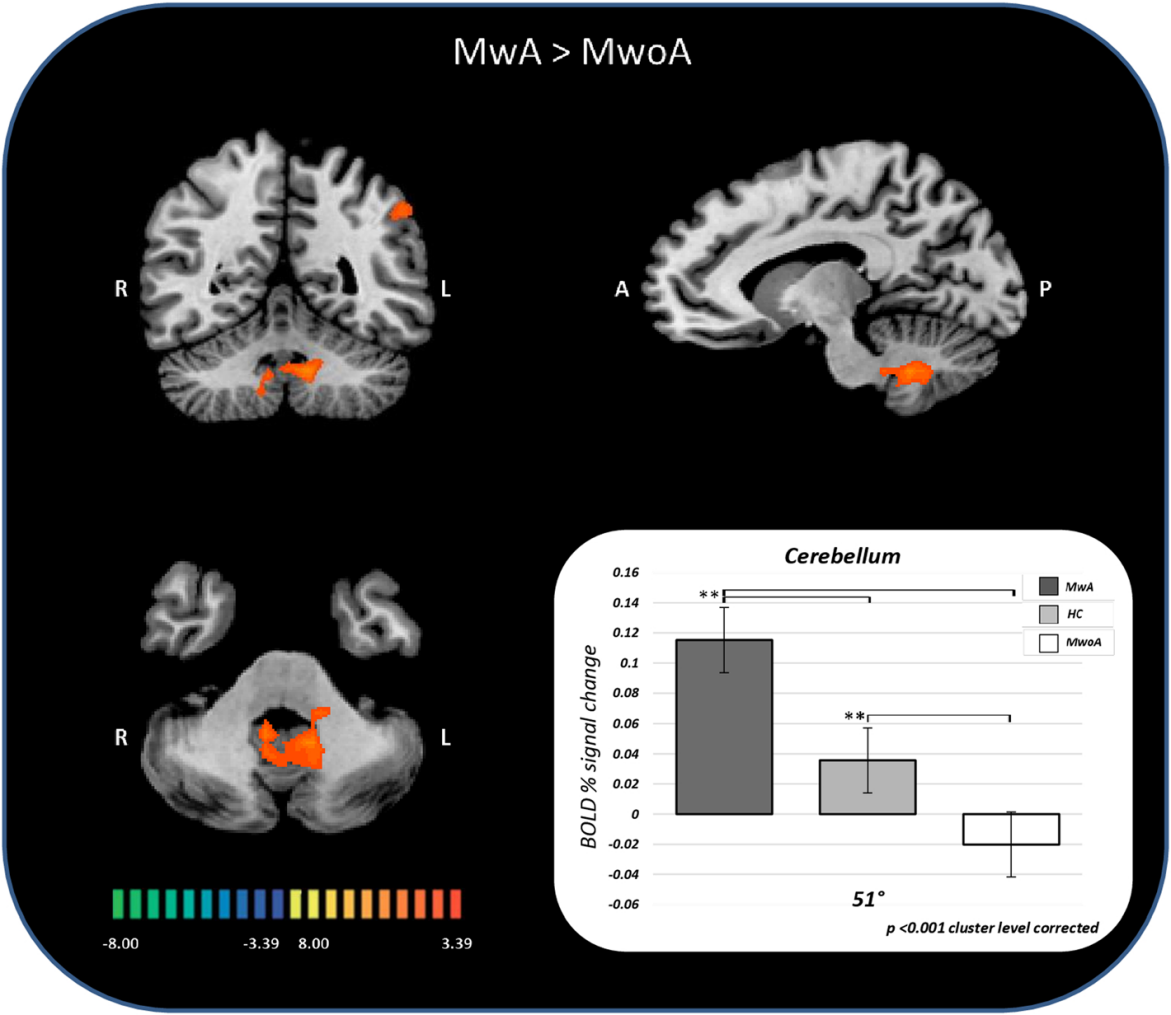
Percent of BOLD signal change (mean ± SE) at 51 °C from whole brain analysis in group comparisons between patients with MwA, patients with MwoA and HC. SE = standard error; MwA = migraine with aura; MwoA = migraine without aura; HC = healthy controls.

When comparing patient groups among them and with HC, during moderately painful THS (51°), we observed: i) a significantly greater BOLD-response in left lingual gyrus, inferior parietal lobe, inferior frontal gyrus (see Figs. 1 and 2) and cerebellum in patients with MwA compared to MwoA patients (Fig. 3); ii) a significantly greater BOLD-response in the cerebellum in patients with MwA compared to HC (Fig. 3); iii) a significantly greater BOLD-response in medial frontal gyrus (see Figs. 1 and 2) in patients with MwA compared to HC; iv) no other difference in BOLD response has been observed between both MwA patients group and MwoA patients group and HC.

Altogether, lingual gyrus, inferior parietal lobule, inferior frontal gyrus and medial frontal gyrus encompassing the so-called advanced visual processing (AVN) [10–13].

**Fig. 4:**
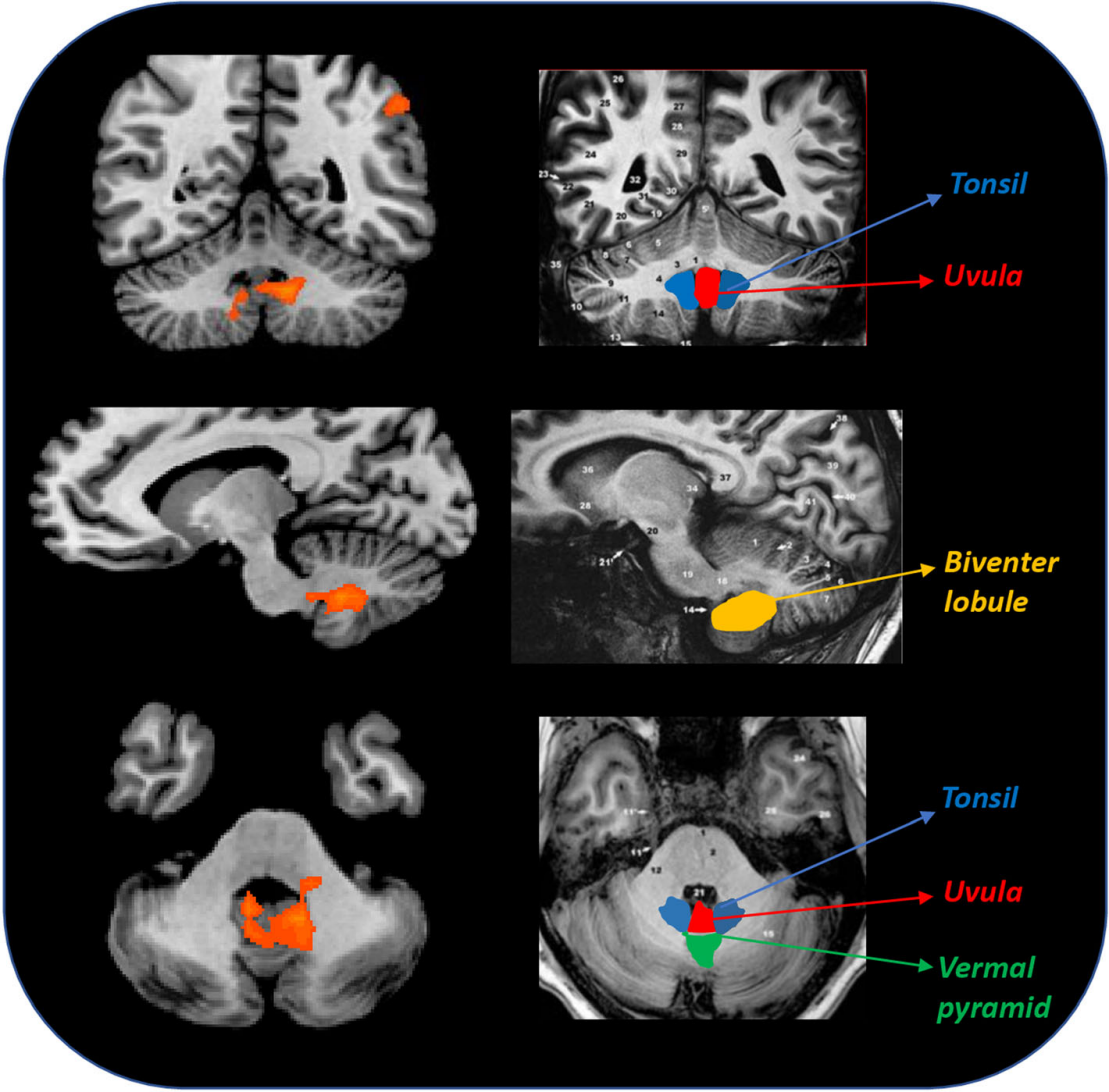
Correspondence of function and structure of midline and lower structures of the cerebellum showing interictal increased response to THS in MwA patients. The coloured areas highlight the uvula, the tonsils and the biventer lobule. (adapted from Duvernoy HM, 1995). THS: trigeminal heat stimulation; MwA = migraine with aura.

Talairach coordinates of significant cluster differences between groups are reported in Table 2. Mean values of percentage BOLD signal changes were extracted from following clusters: lingual gyrus (MwA: 0.12 ± 0.03; HC: 0.031 ± 0.02; MwoA: − 0.029 ± 0.02; MwA vs HC p:0.005, MwA vs MwoA *p* < 0.001, MwoA vs HC p:0.02), inferior parietal lobule (MwA: 0.11 ± 0.02; HC: 0.05 ± 0.03; MwoA: − 0.03 ± 0.05; MwA vs HC p:0.06, MwA vs MwoA *p* < 0.001, MwoA vs HC p:0.002), inferior frontal gyrus (MwA: 0.13 ± 0.03; HC: 0.06 ± 0.03; MwoA:-0.05 ± 0.02; MwA vs HC p:0.13, MwA vs MwoA *p* < 0.001, MwoA vs HC p:0.001), medial frontal gyrus (MwA: 0.13 ± 0.03; HC: 0.01 ± 0.02; MwoA:0.04 ± 0.02; MwA vs HC p: *p* < 0.001, MwA vs MwoA p:0.008, MwoA vs HC p:0.39) and cerebellum (MwA: 0.11 ± 0.02; HC: 0.03 ± 0.01; MwoA: − 0.02 ± 0.01; MwA vs HC *p* < 0.001, MwA vs MwoA *p* < 0.001, MwoA vs HC p:0.006) (see Figs. 2, 3 and 4).

**Table 2:** Significant cluster differences between patients with MwA, patients with MwoA and HC (*p* < 0.001 cluster level corrected)

THS at 51 °C	Anatomical label	BA	Talairach coordinates	t-value
MwA > MwoA	Cerebellum		−9;-52;-29	5.376
MwA > HC				
MwA > MwoA	L lingual gyrus	17	0;-91;1	5.227
MwA > MwoA	L inferior parietal lobule	40	−45;-55;43	4.511
MwA > MwoA	L inferior frontal gyrus	9	−36;8;28	4.480
MwA > HC	L medial frontal gyrus	8	−6;38;37	4.411

*THS* trigeminal heat stimulation, *MwA* patients with migraine with aura, *MwoA* patients with migraine without aura, *HC* healthy controls, *BA* Brodmann area, *L* left, *R* right

### Correlation analyses

There were no statistically significant correlations between any clinical parameters of migraine severity and BOLD signal changes during mildly painful THS (41°) and moderately painful THS (51 °C) in patients with MwoA. In the group of MwA patients we observed a significant positive correlation between cerebellar BOLD response to moderately painful (51°) THS and disease duration (*r* = 0.53; *p* = 0.03) (i.e., the longer the history of migraine in years, the higher the cerebellar activation). However, when a more conservative *p*-value threshold (Bonferroni correction) was used, no statistically significant association could be found between fMRI signals and disease duration. Interestingly, there were no statistically significant correlations between perceived pain intensity (by NRS) and BOLD-fMRI signal changes at any level of experimental stimuli in migraine patients (both MwoA and MwA) and HC.

## Discussion

In the present study, using a whole-brain BOLD-fMRI approach we found, consistently with previous observations, that THS activates cortical and subcortical areas, known to be involved in central pain processing [9]. While all participants demonstrated this general pattern of response, patients with MwA exhibited, during moderately painful stimuli, an increased activation of higher cortical areas constituting a known distributed network associated with AVN, including lingual gyrus, inferior parietal lobule, inferior frontal gyrus and medial frontal gyrus [10–13]. Moreover, a significantly greater cerebellar activation was observed in patients with MwA compared to both HC and MwoA. Interestingly, the magnitude of cerebellar activation was correlated with disease duration of patients with MwA. However, pain intensity ratings were not significantly different between the three groups at any level of THS.

In the last decades, based on the prominent role played by visual symptoms experienced by patients with MwA, visual pathways have been extensively explored to identify whether the visual pathways hyperexcitability could subtend both the hyperresponsiveness to visual stimuli and the increased propensity to experience aura or, contrarily, whether the abnormal visual processing associated with aura phenomenon leads to brain hyperresponsiveness to visual stimuli [14].

Advances in functional neuroimaging techniques have dramatically improved our insight of mechanisms underlying aura phenomenon in migraine patients. Among these, visual pathways activation has been observed in patients with MwA, suggesting that cortical hyperresponsiveness in migraine is directly related to the presence of aura in these patients [15]. More recently, in a multiparametric fMRI study, a stronger functional connectivity in visual network (specifically in lingual gyrus) has been demonstrated in patients with MwA compared to HC and patients with MwoA, in absence of structural or microstructural abnormalities in the same brain areas [16]. These data are well-fitting with abnormal levels of GABA and glutamate observed by magnetic resonance spectroscopy in the primary visual cortex of MwA patients during visual stimulation [17].

However, despite the growing body of literature exploring the neural correlates of both aura and headache phases during the migraine cycle [18, 19], only few studies have specifically assessed brain activity in patients with MwA during experimental moderately painful stimulations. Among these, two seminal PET studies showed the peculiar reciprocal interactions between pain experience and aura phenomenon in migraine patients [20, 21]. Specifically, these findings have demonstrated that visual pathway activation by luminous stimuli was potentiated by trigeminal nociception, strongly suggesting a “visual network-pain network” integration in these patients.

The present results, in line with above-mentioned observations show, for the first time, the hyperresponsiveness of the AVN during experimental moderately painful stimulations, in patients with MwA when compared to patients with MwoA and HC.

The AVN, encompassing areas of the dorsal processing stream and fronto-lateral regions, is relevant for processing the spatial attributes of visual information, memory-guided saccades, spatial working memory and the executive control of spatial attention, as well as in different types of saccadic eye movements as demonstrated by several studies in human and non-human primates [11–13, 22, 23]. The hypothesis that neural mechanisms subtending aura phenomenon may be strictly correlated with specific hyperresponsiveness of brain areas involved in AVN has been recently supported [10]. Indeed, in the same cortical areas an increased BOLD response to visual stimulation has been observed in patients with MwA, with side-fixed visual aura attacks, in hemispheres involved by aura phenomenon when compared with the hemisphere not involved by aura phenomenon in the same patients (even during the interictal phase) and HC [10]. Finally, the involvement of the medial frontal gyrus has been observed in structural and functional studies in patients with migraine, specifically in those experiencing aura phenomenon [24, 25].

Beside the activation of AVN, we observed a significantly increased BOLD-response to THS in the midline and lower structures of the cerebellum (e.g.: uvula, tonsils and biventer lobule) in patients with MwA when compared with both patients with MwoA and HC. Although its role has been previously underestimated in human pain perception, it is now well-known that cerebellum is involved in pain processing [26, 27] as demonstrated also by altered experimental pain perception after cerebellar infarction [28]. More specifically, during trigeminal nociception, the activation of specific cerebellar areas as well as their functional connections with both the descending anti-nociceptive network and cortical hubs of pain processing have been observed [29].

Based on these observations, several structural and functional studies have been conducted in the attempt to clarify the putative role played by the cerebellum in migraine [30]. Among these, an increased activity of the cerebellar crus with a concomitant decreased functional connectivity with thalamus have been demonstrated during trigeminal stimulations. The findings suggest a recruitment of cerebellar additional resources to overcome the diminished functional cerebellar-thalamic connectivity in patients with MwoA [30]. However, the cerebellar compensatory mechanism seems to be ineffective, inducing a dysfunctional thalamic gating of external stimuli addressed to cortical areas and leading to susceptibility for migraine attacks [31]. Interestingly, a reduced cerebellar inhibitory control on cerebral cortex has been supported also in patients with MwA, using a transcranial magnetic stimulation protocol [32].

The increased cerebellar activity founded in the present study suggest the same compensatory and ineffective, in other word “maladaptive”, mechanism in patients with MwA during trigeminal nociceptive experience.

Considering our cortical and cerebellar findings, based on the well-known connections between cerebellar structures (e.g. pyramid, uvula, tonsils and biventer lobule) and AVN [33, 34] we speculate that the AVN hyperresponsiveness to THS might be due to the lack of cerebellar inhibitory control on thalamic sensory gating and, thus, on the brain cortex.

Our BOLD-fMRI cortical (e.g.: lingual gyrus, inferior parietal lobule, inferior frontal gyrus and medial frontal gyrus) findings showed no correlations with MwA clinical features.

Finally, although previous data supported that the activity of the cerebellum is modulated by the perceived intensity of pain, the increased activity of cerebellum and AVN does not affect pain intensity perception in patients with MwA, being the pain ratings not significantly different between the three study groups, consistently with previous experimental data [6, 7, 35].

Taken together, our findings suggest that MwA may be characterized by a peculiar hyperresponsiveness of the distributed AVN not only to visual, as previously demonstrated [10] but also to moderately painful trigeminal stimulations, suggesting a “visual-pain networks integration”, likely by means of a “maladaptive” activity of cerebellum.

Finally, our secondary analyses did not show statistically significant association between BOLD-fMRI response and clinical parameters of disease severity except for disease duration, showing statistically significant association with BOLD-fMRI response not surviving when a more conservative *p*-value threshold (Bonferroni correction) has been used. Thereby, we believe that visual aura phenotype per se could justify the observed increased activity in higher cortical areas of the network associated with advanced visual processing and in the cerebellum in patients with migraine with aura, as previously demonstrated also for resting state visual network functional connectivity [16].

Our study is not exempt from some limitations. First of all, we cannot completely exclude the putative effect of habituation or sensitisation phenomenon due to repetitive THS on our findings, despite pain intensity ratings were not changed in the course of experimental stimulations, the random modality of the THS and the well-known absence of habituation in patients with migraine. Moreover, we cannot compare the AVN response to THS with response to visual stimulations (not included in the experimental paradigm). In addition, patients with MwoA and MwA experienced different frequency of attacks. On the other hand, because this is general to the migraine population, we believe that it may give us the opportunity to evaluate the “natural history and phenotype” of the two migraine subtypes.

## Conclusion

In conclusions, we suggest that mechanisms underlying MwA could affect sensory integration between trigeminal pain network and visual network, adding new stimulating pathophysiological interpretations to the model of migraine as a “dysfunction of neurolimbic pain network”.^35^ Indeed, we propose to enlarge this concept in favour of a more comprehensive “dysfunction of neurolimbic-visual-pain network” model in MwA patients, characterized by a functional correlation between pain-modulating circuits not only with the cortical limbic centers [36] but with the AVN as well.

## Additional file

Additional file 1: Absolute movement in the 3-D coordinate system during the fMRI scan sessions and average heart and respiratory rates for each subjects group. (DOCX 17 kb)

## Abbreviations

AVN: Advanced visual processing
BOLD: Blood oxygen level-dependent
CHEPS: Contact heat evoked potential stimulator
CSD: Cortical spreading depression
fMRI: Functional magnetic resonance imaging
GLM: General-linear-model
HC: Healthy controls
HIT-6: Headache impact test 6
MIDAS: Migraine disability assessment scale
MwA: Migraine with aura
MwoA: Migraine without aura
NRS: Numerical rating scale
THS: Trigeminal heat stimulation
