# Supplementary material for: Advanced visual network and cerebellar hyperresponsiveness to trigeminal nociception in migraine with aura
Source: J Headache Pain. 2019 May 3;20(1):46. doi: 10.1186/s10194-019-1002-3 (PMC6734311; doi:10.1186/s10194-019-1002-3)
Supplement: Supplementary file 1 — Absolute movement in the 3-D coordinate system during the fMRI scan sessions and average heart and respiratory rates for each subjects group. (DOCX 17 kb) [file 10194_2019_1002_MOESM1_ESM.docx]

Additional file 1

**Table S1**. Absolute movement in the 3-D coordinate system during the fMRI scan sessions

| Movement | Group | Mean ± SD | *p* value |
| --- | --- | --- | --- |
| T-x | MwoA | 0.42 ± 0.35 | 0.36^a^ |
|  | MwA | 0.32 ± 0.27 | 0.92^b^ |
|  | HC | 0.33 ± 0.37 | 0.49^c^ |
|  |  |  |  |
| T-y | MwoA | 0.24 ± 0.19 | 0.90^a^ |
|  | MwA | 0.23 ± 0.20 | 0.44^b^ |
|  | HC | 0.18 ± 0.19 | 0.34^c^ |
|  |  |  |  |
| T-z | MwoA | 0.91 ± 0.73 | 0.32^a^ |
|  | MwA | 0.69 ± 0.56 | 0.71^b^ |
|  | HC | 0.78 ± 0.74 | 0.61^c^ |
|  |  |  |  |
| R-x | MwoA | 0.98 ± 0.85 | 0.07^a^ |
|  | MwA | 0.53 ± 0.39 | 0.69^b^ |
|  | HC | 0.66 ± 1.21 | 0.40^c^ |
|  |  |  |  |
| R-y | MwoA | 0.49 ± 0.39 | 0.44^a^ |
|  | MwA | 0.39 ± 0.34 | 0.84^b^ |
|  | HC | 0.42 ± 0.51 | 0.69^c^ |
|  |  |  |  |
| R-z | MwoA | 0.48 ± 0.34 | 0.09^a^ |
|  | MwA | 0.31 ± 0.22 | 0.21^b^ |
|  | HC | 0.48 ± 0.47 | 0.99^c^ |

T: translation; R: rotation; ^a^ MwoA vs MwA; ^b^ MwA vs HC; ^c^ MwoA vs HC. MwoA: patients with migraine without aura; MwA: patients with migraine with aura; HC: healthy controls.

**Table S2. Average heart and respiratory rates for each subjects group**

| Parameter | Group | Mean ± SD | *p* value |
| --- | --- | --- | --- |
| Hearth rate | MwoA | 70.82 ± 5 | 0.21^a^ |
|  | MwA | 68.89 ± 4.1 | 0.66^b^ |
|  | HC | 70.13 ± 3.74 | 0.37^c^ |
|  |  |  |  |
| Respiratory rate | MwoA | 13.59 ± 1.22 | 0.93^a^ |
|  | MwA | 13,55 ± 1.2 | 0.86^b^ |
|  | HC | 13.66 ± 1.23 | 0.79^c^ |

^a^MwoA vs MwA; ^b^MwA vs HC; ^c^MwoA vs HC. MwoA= patients with migraine without aura; MwA= patients with migraine with aura; HC= healthy controls.
